# Factors associated with social support among older inpatients with schizophrenia: a cross-sectional study from a tertiary psychiatric hospital in Guangzhou, China

**DOI:** 10.3389/fpubh.2026.1915408

**Published:** 2026-09-03

**Authors:** Jinrong Li, Junrong Ye, Jiao Chen, Dingjie Liu, Xueyu Zheng, Yulin Li, Na Ma, Yuhan Zhang, Yanheng Wei, Tian Zhou, Jianxiong Guo, Aixiang Xiao

**Affiliations:** 1 Department of Nursing Administration, The Affiliated Brain Hospital, Guangzhou Medical University, Guangzhou, China; 2 Key Laboratory of Neurogenetics and Channelopathies of Guangdong Province and the Ministry of Education China, Guangzhou Medical University, Guangzhou, China; 3 Department of Traditional Chinese Medicine, Affiliated Brain Hospital of Guangzhou Medical University, Guangzhou, China; 4 School of Nursing, Guangzhou Medical University, Guangzhou, China

**Keywords:** cross-sectional study, older patients, schizophrenia, social support, social support rating scale

## Abstract

**Background:**

Older inpatients with schizophrenia have lower social support levels that may affect clinical outcomes, yet the associated factors in this population remain underexplored.

**Objective:**

To explore the factors associated with social support in older inpatients with schizophrenia.

**Methods:**

A cross-sectional study was conducted among 204 inpatients with schizophrenia recruited from a tertiary psychiatric hospital in Guangzhou, China, between February 2023 and December 2024. Social support and its three dimensions were assessed using the Social Support Rating Scale (SSRS). Pearson correlation analysis and multiple linear regression were applied to identify factors associated with social support.

**Results:**

The mean SSRS total score was 30.26 ± 7.07. Multiple linear regression revealed that higher levels of insight (ITAQ: *β* = 0.431, 95% CI: 0.308 ~ 0.555, *p* < 0.001) and having a spouse (*β* = 4.393, 95% CI: 2.563 ~ 6.222, *p* < 0.001) were positively associated with higher SSRS total scores, whereas higher suicide risk (NGASR: *β* = −0.507, 95% CI: −0.848 ~ −0.166, *p* = 0.004), greater depressive symptoms (GDS: *β* = −0.156, 95% CI: −0.282 ~ −0.030, *p* = 0.016), and longer course of schizophrenia (*β* = −0.092, 95% CI: −0.154 ~ −0.029, *p* = 0.004) were negatively associated. The regression models explained 36.3% of the variance (adjusted *R*^2^ = 0.323, *F* = 9.019, *p* < 0.001).

**Conclusion:**

Social support among older inpatients with schizophrenia is associated with marital status, insight level, suicide risk, depressive symptoms, and disease duration. Multidimensional interventions targeting specific support dimensions are needed to improve social support in this population.

## 1 Introduction

Schizophrenia is a chronic mental disorder affecting more than 23 million individuals globally (1). Individuals aged 55 years and above had been projected to account for 25% or more of the total population of patients with schizophrenia worldwide due to the aging population (2). Despite a decline in new cases among older adults in China, the crude DALY rate for schizophrenia increased from 202.85/100,000 in 1990 to 258.54/100,000 in 2019 (a 27.4% increase) (3). This growing demand for healthcare services poses severe challenges to the healthcare system in China. Previous studies have shown that, in addition to medical resources, social support systems may also constitute a key variable influencing the prognosis of schizophrenia (4).

Social support, referring to the emotional, material, informational, and other forms of assistance provided by others or social networks, serves as a crucial resource for individuals coping with various disease-related challenges (5). While current research has recognized the positive role of social support in schizophrenia treatment and rehabilitation (6, 7), some theoretical limitations should be noted. Social support has frequently been used as an outcome indicator in intervention studies, as it can be readily quantified using standardized scales routinely incorporated into efficacy evaluations (8). Additionally, existing assessments of social support have tended to emphasize objective support, while greater attention to other dimensions is warranted (9).

However, previous studies on social support in schizophrenia have tended to focus on younger or community-dwelling populations (6, 10). The social support dynamics of older inpatients was fundamentally different due to prolonged hospitalization, restricted social contact, age-related cognitive decline, and the clinical context of involuntary treatment (11). Furthermore, while individual factors such as marital status, insight, and depressive symptoms had been examined separately, few studies simultaneously examined the role of key clinical and psychosocial factors, including suicide risk, depressive symptoms, insight, disease duration, and caregiver support in shaping social support among this specific population (12, 13). Understanding these associations is essential for identifying the most vulnerable subgroups and developing targeted discharge planning and psychosocial interventions in clinical practice.

Several clinical factors may be particularly relevant to social support in this context. First, up to half of patients with schizophrenia manifest suicidal behaviors during hospitalization, and social isolation resulting from low social support has been identified as a contributing factor to these behaviors (14). In China, the prevalence of suicide attempts among older patients with schizophrenia was 15.2%, with depression as a significant risk factor (15). Although enhanced social support has been shown to reduce suicide risk and alleviate depressive symptoms, the direction of these relationships may be bidirectional: psychological distress may also impair patients’ ability to maintain social relationships and to seek or utilize available support (10). This is particularly concerning in older patients, as age-related cognitive decline may further exacerbate the negative impact of such distress on social support perception and utilization. Second, patients with schizophrenia commonly present with varying degrees of insight impairment, which may also affect their ability to obtain and maintain social support by influencing treatment adherence, communication effectiveness, and social initiative (16). Although the relationship between insight and social support has been examined in younger populations (17), it remains poorly understood among older inpatients, whose cognitive decline and evolving support sources may amplify or modify this association. Third, longer disease duration may erode social support networks through progressive social withdrawal, accumulated stigma, and loss of social roles during prolonged institutionalization (18). Finally, the type of primary caregiver, specifically whether patients depend on others for daily care or are independent in self-care, may determine the amount of tangible support they receive during hospitalization. However, these factors have received limited attention in previous studies on social support in older inpatients with schizophrenia.

Despite the potential clinical relevance of these factors in shaping social support among older inpatients with schizophrenia, few studies have simultaneously examined them in a single analytical framework. Therefore, this study aimed to: (1) assess the level of social support and its three dimensions among older inpatients with schizophrenia; (2) examine the correlations between social support and these clinical variables; and (3) identify factors independently associated with social support using multiple linear regression.

## 2 Methods

### 2.1 Participants and data collection

A consecutive sampling method was used to recruit patients admitted to a public psychiatric hospital in Guangzhou from February 2023 to December 2024. The inclusion criteria were as follows: (1) a diagnosis of schizophrenia according to the International Classification of Diseases 10th Revision (ICD-10); (2) age ≥ 50 years. This threshold was chosen because patients with schizophrenia often experience premature aging and earlier physical comorbidity, and age ≥ 50 is commonly used in geriatric psychiatry research to define older adults with schizophrenia (11). This threshold has also been used in recent Chinese studies of older patients with schizophrenia (15). (3) willingness to provide written informed consent.

Exclusion criteria: (1) patients with end-stage diseases (life expectancy < 6 months); (2) patients with hearing or visual impairment that might hinder communication; (3) unwillingness to complete the investigation for any reason.

Written informed consent was obtained from each participant or, when the participant lacked decision-making capacity, from their legal guardian.

### 2.2 Sample size estimation

The sample size was estimated using PASS 21 software for a multiple linear regression. Considering the candidate variables anticipated to be entered into the regression model and a 20% dropout rate, the minimum required sample size was calculated as 135. Ultimately, 204 participants were enrolled, resulting in a ratio of approximately 17 participants per variable, which exceeded the minimum requirement.

### 2.3 Data collection procedure

**Figure 1:**
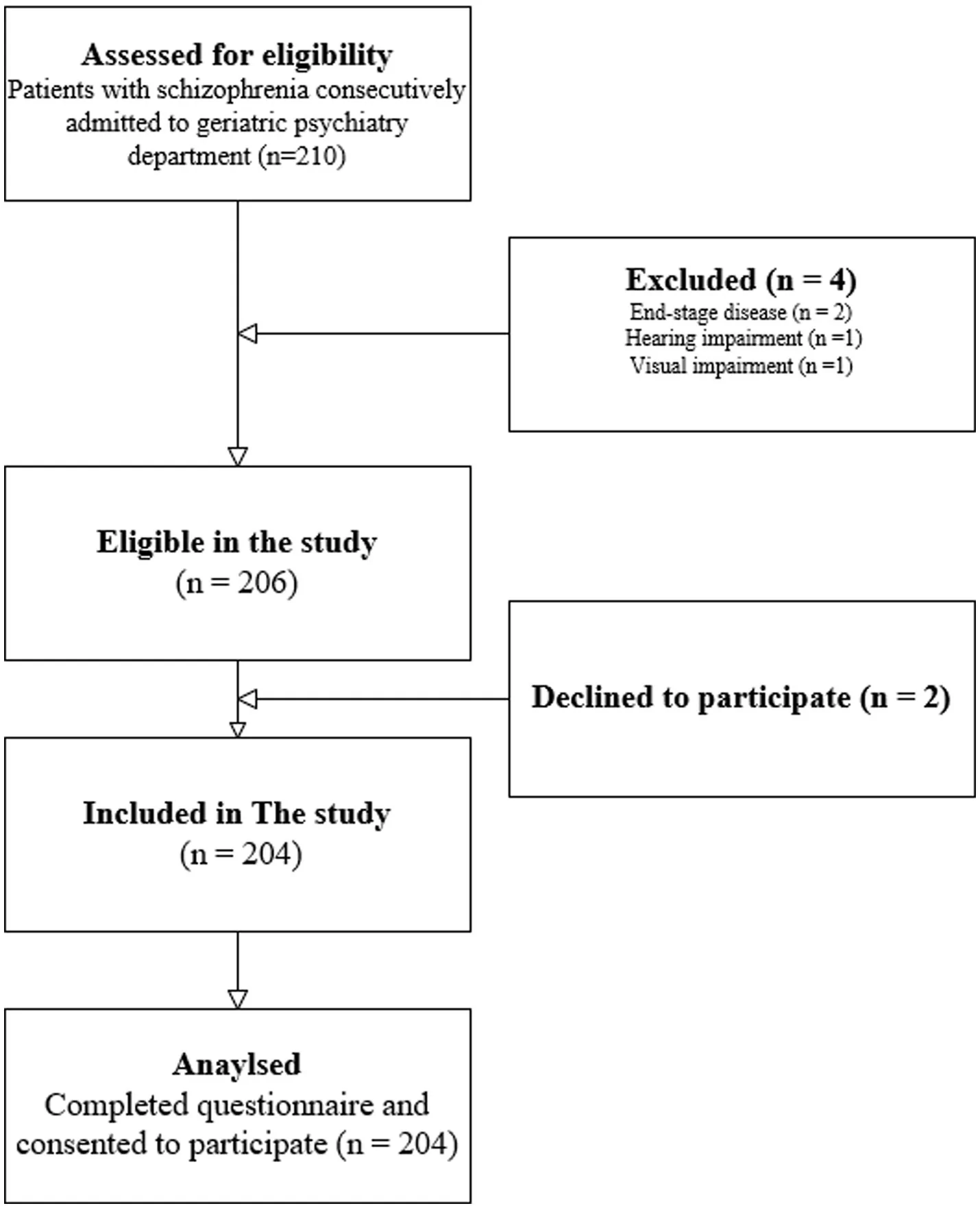
Participant flow diagram.

Data were collected at two time points. Baseline demographic, clinical, and psychosocial data, including scores on the SSRS, ITAQ, GDS-30, BPRS, and NGASR, were collected within three days of admission. Clinical variables pertaining to the entire hospitalization, including total length of stay, use of physical restraint, receipt of modified electroconvulsive therapy (MECT), and number of antipsychotic medications prescribed, were extracted from medical records at discharge. Trained research assistants conducted semi-structured interviews in Mandarin or the local dialect. Because older patients may have difficulty completing self-report questionnaires due to declines in vision or cognitive function, appropriate guidance and explanations were provided to improve both response quality and data completeness. All research assistants underwent standardized training and consistency testing before data collection. The questionnaires were distributed by trained research assistants; after the participants completed them, the research assistants collected the questionnaires and checked the responses for completeness. During the study period, a total of 210 patients with schizophrenia were consecutively admitted to the geriatric psychiatry department. After screening, 4 patients were excluded according to the exclusion criteria (2 with end-stage disease, 1 with hearing impairment, and 1 with visual impairment), leaving 206 eligible patients. Of the 206 eligible patients, 2 declined to participate, and the remaining 204 provided written informed consent and completed the questionnaires. The participation rate among eligible patients was 99.0% (204/206), and the questionnaire completion rate among enrolled participants was 100% (204/204) (Figure 1). The high participation and completion rates were attributable to the supervised inpatient setting, where trained research assistants provided one-on-one assistance and checked the returned questionnaires for completeness immediately. Cognitive impairment was not an exclusion criterion; patients with cognitive or sensory difficulties who could communicate were included and received appropriate guidance and explanations. For patients who lacked decision-making capacity, written informed consent was obtained from their legal guardians, and in a small number of cases guardians also assisted the patients in completing the questionnaires (*n* = 2).

### 2.4 Measurements

#### 2.4.1 General information questionnaire

A self-designed questionnaire was used to collect demographic and clinical data, including: gender, age, educational level, marital status (having a spouse or not), primary caregiver (self-care or dependent on caregivers), course of schizophrenia (years since first diagnosis, as documented in medical records), emergency admission (yes/no), physical restraint during hospitalization (yes/no), receipt of modified electroconvulsive therapy during the current hospitalization (MECT, yes/no), number of antipsychotic medications used during hospitalization (none, monotherapy, polypharmacy), and total duration of hospitalization (days).

#### 2.4.2 Social Support Rating Scale (SSRS)

The Chinese version of the Social Support Rating Scale (SSRS) was developed by Shuiyuan Xiao in 1986. The scale includes three dimensions: objective support, subjective support, and the utilization of social support. Scores range from 13 to 66; a total score of less than 22 indicates low social support, between 23 and 44 indicates medium social support, and more than 45 indicates high social support (19). The SSRS has been widely utilized across diverse Chinese populations and has demonstrated good reliability and validity. In the present study, the Cronbach’s alpha was 0.710. In the present study, the SSRS was self-completed by the participants, with trained research assistants providing reading and explanatory assistance when needed.

#### 2.4.3 Nurses’ global assessment of suicide risk (NGASR)

The NGASR was developed by Cutcliffe and Barker in 2004 to measure suicide risk. This scale comprises 15 items, each assigned a specific point value based on their relevance to suicide risk. Scores range from 0 (low suicide risk) to 25 (high suicide risk) (20). In the present study, the NGASR was rated by trained research assistants based on a structured clinical interview and clinical observation.

#### 2.4.4 Insight and treatment attitudes questionnaire (ITAQ)

The ITAQ, developed by McEvoy in 1989, consists of 11 items divided into two main sections: awareness of illness and attitude toward treatment. Scores range from 0 (no insight) to 22 (full insight), with higher scores indicating a greater level of insight and more positive attitudes toward treatment (21). The ITAQ was administered by trained research assistants as a structured interview.

#### 2.4.5 Geriatric depression scale 30 items (GDS-30)

The GDS-30 Chinese version was utilized to identify depressive symptoms in older adults. The total score ranges from 0 to 30, with higher scores indicating more severe depression. The GDS has been extensively validated across different populations in China (22). In the present study, the GDS-30 was self-completed by the participants, with trained research assistants providing reading and explanatory assistance when needed.

#### 2.4.6 Brief psychiatric rating scale (BPRS)

The BPRS was used to assess the severity of psychiatric symptoms. The BPRS comprises 18 items rated on a 7-point Likert scale (1 = not present to 7 = extremely severe), yielding a total score ranging from 18 to 126, with higher scores indicating greater symptom severity. The Chinese version of the BPRS has demonstrated good reliability and validity among patients with schizophrenia in China (23). In the present study, the BPRS was rated by trained research assistants based on a structured clinical interview and clinical observation.

### 2.5 Statistical analysis

Data were analyzed using SPSS version 26.0. Descriptive statistics were presented as mean ± standard deviation (SD) for continuous variables and as frequencies and percentages for categorical variables.

Group comparisons were performed using independent-sample t-tests or one-way ANOVA for normally distributed continuous variables, and the Mann–Whitney U test or Kruskal-Wallis test for non-normally distributed variables. Normality was assessed using the Shapiro–Wilk test, and homogeneity of variances was evaluated using Levene’s test.

Pearson or Spearman correlation analyses were conducted to examine the associations among SSRS total and dimensional scores, BPRS, ITAQ, GDS-30, NGASR, course of schizophrenia, and total duration of hospitalization.

Candidate variables were entered into the multiple linear regression models based on clinical relevance and prior literature, regardless of their statistical significance in univariate analyses. All regression models included the same set of candidate variables: age, sex, educational level, course of schizophrenia, BPRS, ITAQ, GDS-30, NGASR, marital status, emergency admission, and self-care status. Categorical variables were coded as: sex (0 = female, 1 = male); marital status (0 = no spouse, 1 = having a spouse); self-care status (0 = dependent on caregivers, 1 = self-care); emergency admission (0 = no, 1 = yes); educational level (1 = primary school and below, 2 = middle school, 3 = senior high school and above, reference: primary school and below). Assumption tests included residual normality (Shapiro–Wilk), homoscedasticity, multicollinearity (VIF), and influential cases (Cook’s distance). There were no missing item-level data in the returned questionnaires; therefore, no missing data imputation was applied. A two-tailed *p* < 0.05 was considered statistically significant.

## 3 Results

### 3.1 Sociodemographic characteristics

A total of 204 older inpatients with schizophrenia were included. The mean age was 64.16 ± 7.79 years, with the majority aged 60–69 years (47.1%). Females comprised 74.5% of the sample. Most participants had a spouse (64.2%) and relied on caregivers (80.9%). Regarding educational level, 30.4% had primary education or below, 34.3% had completed middle school, and 35.3% had senior high school or above.

The mean disease duration was 18.07 ± 13.68 years, with 78.4% having a disease course longer than 5 years. During hospitalization, 45.1% of patients had a history of emergency admission, 6.4% had experienced physical restraint, and 3.9% had received MECT during the current hospitalization. Regarding the number of antipsychotic medications prescribed, 9.3% of patients received none, 39.2% received monotherapy, and 51.5% received polypharmacy. The mean BPRS score was 34.76 ± 11.22, and the mean total hospitalization duration was 27.00 ± 12.74 days (Table 1).

**Table 1:** Participant characteristics and comparison of SSRS score between participants with different characteristics (*n* = 204).

Variables	Participants		Total SSRS score			Objective support			Subjective support			Utilization of social support		
	*n*	Percentage (%)	M ± SD	*t/F/Z*	*p*	M ± SD	*t/F/Z*	*p*	M ± SD	*t/F/Z*	*p*	M ± SD	*t/F/Z*	*p*
Social Support Rating Scale (SSRS)			30.26 ± 7.07			8.60 ± 2.55			16.72 ± 4.50			4.94 ± 2.15		
Gender														
Male	52	25.5	30.19 ± 7.38	0.403	0.526	8.90 ± 2.90	0.872	0.351	16.60 ± 4.53	0.020	0.888	4.69 ± 2.15	0.038	0.846
Female	152	74.5	30.34 ± 6.98			8.49 ± 2.41			16.79 ± 4.48			5.06 ± 2.16		
Age (years), M ± SD 64.16 ± 7.79														
<60 years old	60	29.4	30.25 ± 6.773	0.992	0.373	8.50 ± 2.77	0.377	0.687	16.42 ± 4.31	0.829	0.438	5.33 ± 2.67	3.692	0.158
60 ~ 69 years old	96	47.1	29.73 ± 7.07			8.51 ± 2.42			16.57 ± 4.68			4.65 ± 1.98		
≥70 years old	48	23.5	31.45 ± 7.39			8.86 ± 2.50			17.43 ± 4.30			5.16 ± 2.31		
Educational level														
Primary and below	62	30.4	31.38 ± 6.19	0.347	0.707	9.03 ± 1.83	0.456	0.796	17.55 ± 4.11	0.903	0.637	4.80 ± 2.06	2.143	0.120
Middle school	70	34.3	29.37 ± 7.24			8.44 ± 2.82			15.84 ± 4.72			5.09 ± 2.13		
Senior high school and above	72	35.3	30.25 ± 7.37			8.41 ± 2.72			16.85 ± 4.31			5.00 ± 2.32		
Marital status (having a spouse or not)														
Yes	131	64.2	31.79 ± 6.79	**4.301**	**<0.001**	9.31 ± 2.33	**5.733**	**<0.001**	17.57 ± 4.46	**3.740**	**<0.001**	4.90 ± 2.10	−0.315	0.753
No	73	35.8	27.52 ± 6.79			7.33 ± 2.44			15.19 ± 4.17			5.00 ± 2.26		
Self-care status														
Self-care	39	19.1	28.36 ± 8.41	−1.628	0.110	7.18 ± 2.75	**−4.020**	**<0.001**	16.41 ± 5.26	−0.478	0.633	4.77 ± 2.02	−0.538	0.591
Dependent on caregivers	165	80.9	30.71 ± 6.68			8.94 ± 2.39			16.79 ± 4.31			4.98 ± 2.19		
Course of schizophrenia (years since first diagnosis, as documented in medical records) M ± SD 18.07 ± 13.68														
≤5	44	21.6	32.66 ± 6.21	**2.574**	**0.011**	9.43 ± 1.84	**2.466**	**0.014**	17.91 ± 4.17	**1.995**	**0.047**	5.32 ± 2.17	1.332	0.184
>5	160	78.4	29.60 ± 7.18			8.38 ± 2.67			16.39 ± 4.54			4.83 ± 2.14		
Physical restraint														
Yes	13	6.4	30.38 ± 5.27	0.066	0.984	9.15 ± 1.46	0.422	0.588	15.85 ± 3.00	1.995	0.047	5.38 ± 2.22	0.776	0.439
No	191	93.6	30.25 ± 7.19			8.57 ± 2.61			16.78 ± 4.58			4.91 ± 2.15		
Emergency admission														
Yes	92	45.1	28.84 ± 6.62	**−2.641**	**0.009**	8.50 ± 2.49	−0.522	0.602	15.80 ± 4.10	**−2.678**	**0.008**	4.53 ± 2.02	**−2.458**	**0.015**
No	112	54.9	31.43 ± 7.26			8.69 ± 2.60			17.47 ± 4.68			5.27 ± 2.21		
Modified electroconvulsive therapy (MECT) during the current hospitalization*														
Yes	8	3.9	30.63 ± 6.14	−0.288	0.774	8.13 ± 2.36	−0.479	0.632	17.50 ± 5.21	−0.429	0.668	5.00 ± 1.93	−0.364	0.716
No	196	96.1	30.24 ± 7.15			8.62 ± 2.56			16.69 ± 4.48			4.93 ± 2.17		
Number of antipsychotics medications														
None	19	9.3	29.89 ± 6.02	0.038	0.963	8.05 ± 2.42	0.682	0.507	16.26 ± 3.77	0.178	0.837	5.58 ± 2.55	1.504	0.225
Monotherapy	80	39.2	30.21 ± 7.65			8.53 ± 2.61			16.63 ± 4.52			5.06 ± 2.24		
Polypharmacy	105	51.5	30.36 ± 6.86			8.76 ± 2.53			16.88 ± 4.63			4.72 ± 2.00		
Baseline disease severity (BPRS), M ± SD				34.76 ± 11.22										
Total duration of hospitalization (days), M ± SD				27.00 ± 12.74										

*MECT comparison used Fisher’s exact test; M ± SD values are descriptive statistics for reference due to small group size. Bold values indicate statistically significant results (*p* < 0.05).

### 3.2 Comparison of SSRS scores between different participant groups

The mean SSRS total score was 30.26 ± 7.07. For the three dimensions, the mean scores were 8.60 ± 2.55 for objective support, 16.72 ± 4.50 for subjective support, and 4.94 ± 2.15 for utilization of social support. Significant differences in SSRS total scores were observed in marital status (*p* < 0.001), course of schizophrenia (*p* = 0.011), and emergency admission status (*p* = 0.009), with higher scores reported by patients who had a spouse, a disease course of less than 5 years, or no emergency admission. For objective support, significant differences were observed in marital status (*p* < 0.001), primary caregiver (*p* < 0.001), and disease course (*p* = 0.014). Regarding subjective support, significant differences were observed in marital status (*p* < 0.001), disease course (*p* = 0.047), physical restraint (*p* = 0.047), and emergency admission (*p* = 0.008). For social support utilization, only emergency admission showed a significant difference (*p* = 0.015). No significant differences were identified across any SSRS dimension for gender, age, educational level, or number of antipsychotic medications (all *p* > 0.05) (Table 1).

### 3.3 Correlation analysis

Table 2 presents the correlation matrix among SSRS scores, clinical scales, and disease-related variables. SSRS total score was significantly positively correlated with ITAQ (*r* = 0.291, *p* < 0.01) and negatively correlated with BPRS (*r* = −0.141, *p* < 0.05), GDS-30 (*r* = −0.152, *p* < 0.05), NGASR (*r* = −0.243, *p* < 0.01), and disease course (*r* = −0.180, *p* < 0.01). However, the correlation between SSRS total score and total hospitalization duration was not statistically significant (*r* = −0.111, *p* > 0.05).

**Table 2:** The correlation of BPRS, ITAQ, GDS-30, NGASR and SSRS among inpatients with schizophrenia (*n* = 204).

Variables	1	2	3	4	5	6	7	8	9	10
1. BPRS	1									
2. ITAQ (levels of insight)	−0.135	1								
3. GDS-30 (levels of depression)	0.330**	0.211**	1							
4. NGASR (risk of suicide)	0.196**	0.236**	0.467**	1						
5. SSRS (social support)	−0.141*	0.291**	−0.152*	−0.243**	1					
6. Objective support	0.040	0.020	−0.140*	−0.165*	0.691**	1				
7. Subjective support	−0.267**	0.259**	−0.191**	−0.250**	0.893**	0.418**	1			
8. Utilization of social support	0.046	0.392**	0.066	−0.079	0.605**	0.213**	0.353**	1		
9. Course of schizophrenia	0.030	0.162*	−0.014	0.083	−0.180**	−0.158*	−0.176*	−0.039	1	
10. Total duration of hospitalization	0.117	−0.124	−0.025	−0.065	−0.111	−0.165*	−0.079	−0.006	0.139*	1

^**^p<0.01, ^*^p<0.05.

Regarding the three dimensions of SSRS: objective support was significantly correlated with GDS-30 (*r* = −0.140, *p* < 0.05), NGASR (*r* = −0.165, *p* < 0.05), disease course (*r* = −0.158, *p* < 0.05), and total hospitalization duration (*r* = −0.165, *p* < 0.05). Subjective support was significantly correlated with BPRS (*r* = −0.267, *p* < 0.01), ITAQ (*r* = 0.259, *p* < 0.01), GDS-30 (*r* = −0.191, *p* < 0.01), NGASR (r = −0.250, *p* < 0.01), and disease course (*r* = −0.176, *p* < 0.05). Utilization of social support was significantly correlated with ITAQ (*r* = 0.392, *p* < 0.01).

### 3.4 Multiple linear regression analysis

Four multiple linear regression models were constructed with SSRS total score, objective support, subjective support, and utilization of social support as dependent variables, respectively. All VIF values were below 2.0, indicating no concerning multicollinearity.

For SSRS total score (Table 3), five variables were independently associated: higher insight level (ITAQ: *β* = 0.431, 95% CI: 0.308 ~ 0.555, *p* < 0.001) and having a spouse (*β* = 4.393, 95% CI: 2.563 ~ 6.222, *p* < 0.001) were positively associated, whereas longer disease duration (*β* = −0.092, 95% CI: −0.154 ~ −0.029, *p* = 0.004), higher suicide risk (NGASR: *β* = −0.507, 95% CI: −0.848 ~ −0.166, *p* = 0.004), and greater depressive symptoms (GDS: *β* = −0.156, 95% CI: −0.282 ~ −0.030, *p* = 0.016) were negatively associated. The model explained 36.3% of the variance (adjusted *R*^2^ = 0.323).

**Table 3:** Linear regression analysis of SSRS total scores in different schizophrenia patients (*n* = 204).

Variables	Non-standardized *β*	Coefficients standardized error	Standardized coefficients beta	*t*	*p*	95% CI	VIF
Constant	23.944	4.230	—	5.660	**<0.001**	15.599 ~ 32.288	—
Gender	−0.942	0.977	−0.059	−0.964	0.336	−2.870 ~ 0.986	1.100
Age	0.090	0.056	0.100	1.614	0.108	−0.020 ~ 0.200	1.145
Course of schizophrenia	−0.092	0.032	−0.178	−2.879	**0.004**	−0.154 ~ −0.029	1.139
Baseline disease severity (BPRS)	0.010	0.041	0.016	0.240	0.811	−0.070 ~ 0.090	1.250
Educational level (Middle school)	0.242	1.059	0.016	0.229	0.819	−1.846 ~ 2.330	1.531
Educational level (Senior high school and above)	0.296	1.075	0.020	0.275	0.784	−1.825 ~ 2.416	1.589
ITAQ (levels of insight)	0.431	0.063	0.459	6.898	**<0.001**	0.308 ~ 0.555	1.318
NGASR (risk of suicide)	−0.507	0.173	−0.202	−2.933	**0.004**	−0.848 ~ −0.166	1.409
GDS (levels of depression)	−0.156	0.064	−0.174	−2.434	**0.016**	−0.282 ~ −0.030	1.525
Marital status (have a spouse)	4.393	0.927	0.300	4.736	**<0.001**	2.563 ~ 6.222	1.197
Emergency admission (Yes)	−1.204	0.878	−0.085	−1.371	0.172	−2.937 ~ 0.528	1.153
Self-care status (self-care)	−1.589	1.113	−0.088	−1.428	0.155	−3.784 ~ 0.605	1.139

R^2^ = 0.363, adjusted R^2^ = 0.323, F = 9.019, *p* < 0.001.

Categorical variable coding: sex (0 = female, 1 = male); educational level (reference: primary school and below); marital status (0 = no spouse, 1 = having a spouse); emergency admission (0 = no, 1 = yes); Self-care status (0 = dependent on caregivers, 1 = self-care). Bold values indicate statistically significant results (*p* < 0.05).

For objective support (Table 4), having a spouse (*β* = 1.888, 95% CI: 1.181 ~ 2.596, *p* < 0.001) and higher insight level (ITAQ: *β* = 0.069, 95% CI: 0.021 ~ 0.117, *p* = 0.005) was positively associated, while self-care (*β* = −1.074, 95% CI: −1.923 ~ −0.225, *p* = 0.013) and higher GDS (*β* = −0.064, 95% CI: −0.112 ~ −0.015, *p* = 0.011) were negatively associated (adjusted *R*^2^ = 0.211).

**Table 4:** Linear regression analysis of SSRS objective social support scores in different schizophrenia patients (*n* = 204).

Variables	Non-standardized β	Coefficients standardized error	Standardized coefficients beta	t	*p*	95% CI	VIF
Constant	4.768	1.636	—	2.914	**0.004**	1.540 ~ 7.996	—
Gender	0.034	0.378	0.006	0.089	0.929	−0.712 ~ 0.779	1.100
Age	0.040	0.022	0.122	1.828	0.069	−0.003 ~ 0.082	1.145
Course of schizophrenia	−0.022	0.012	−0.117	−1.752	0.081	−0.046 ~ 0.003	1.139
Baseline disease severity (BPRS)	0.029	0.016	0.129	1.844	0.067	−0.002 ~ 0.060	1.250
Educational level (Middle school)	0.111	0.410	0.021	0.270	0.787	−0.697 ~ 0.919	1.531
Educational level (Senior high school and above)	0.108	0.416	0.020	0.259	0.796	−0.712 ~ 0.928	1.589
ITAQ (levels of insight)	0.069	0.024	0.204	2.846	**0.005**	0.021 ~ 0.117	1.318
NGASR (risk of suicide)	−0.056	0.067	−0.063	−0.843	0.400	−0.188 ~ 0.076	1.409
GDS (levels of depression)	−0.064	0.025	−0.199	−2.576	**0.011**	−0.112 ~ −0.015	1.525
Marital status (have a spouse)	1.888	0.359	0.360	5.263	**<0.001**	1.181 ~ 2.596	1.197
Emergency admission (Yes)	−0.101	0.340	−0.020	−0.296	0.767	−0.771 ~ 0.570	1.153
Self-care status (self-care)	−1.074	0.430	−0.166	−2.495	**0.013**	−1.923 ~ −0.225	1.139

R^2^ = 0.258, adjusted R^2^ = 0.211, F = 5.511, *p* < 0.001.

Categorical variable coding: sex (0 = female, 1 = male); educational level (reference: primary school and below); marital status (0 = no spouse, 1 = having a spouse); emergency admission (0 = no, 1 = yes); primary caregiver (0 = dependent on caregivers, 1 = self-care). Bold values indicate statistically significant results (*p* < 0.05).

For subjective support (Table 5), higher insight level (ITAQ *β* = 0.222, 95% CI: 0.141 ~ 0.302, *p* < 0.001) and having a spouse (*β* = 2.440, 95% CI: 1.245 ~ 3.635, *p* < 0.001) were positively associated, while longer disease duration (*β* = −0.053, 95% CI: −0.094 ~ −0.012, *p* = 0.012), higher NGASR (*β* = −0.287, 95% CI: −0.510 ~ −0.065, *p* = 0.012) and greater depressive symptoms (GDS: β = −0.091, 95% CI: −0.174 ~ −0.009, *p* = 0.030) were negatively associated (adjusted *R*^2^ = 0.289).

**Table 5:** Linear regression analysis of SSRS subjective social support scores in different schizophrenia patients (*n* = 204).

Variables	Non-standardized *β*	Coefficients standardized error	Standardized coefficients beta	*t*	*p*	95% CI	VIF
Constant	16.079	2.763	—	5.820	**<0.001**	10.629 ~ 21.528	—
Gender	−0.450	0.638	−0.044	−0.704	0.482	−1.709 ~ 0.809	1.100
Age	0.042	0.036	0.073	1.147	0.253	−0.030 ~ 0.114	1.145
Course of schizophrenia	−0.053	0.021	−0.160	−2.531	**0.012**	−0.094 ~ −0.012	1.139
Baseline disease severity (BPRS)	−0.048	0.026	−0.119	−1.801	0.073	−0.100 ~ 0.005	1.250
Educational level (Middle school)	−0.417	0.691	−0.044	−0.603	0.547	−1.781 ~ 0.947	1.531
Educational level (Senior high school and above)	−0.063	0.702	−0.007	−0.090	0.928	−1.448 ~ 1.322	1.589
ITAQ (levels of insight)	0.222	0.041	0.370	5.425	**<0.001**	0.141 ~ 0.302	1.318
NGASR (risk of suicide)	−0.287	0.113	−0.179	−2.545	**0.012**	−0.510 ~ −0.065	1.409
GDS (levels of depression)	−0.091	0.042	−0.160	−2.185	**0.030**	−0.174 ~ −0.009	1.525
Marital status (have a spouse)	2.440	0.606	0.262	4.028	**<0.001**	1.245 ~ 3.635	1.197
Emergency admission (Yes)	−0.978	0.574	−0.109	−1.705	0.090	−2.110 ~ 0.154	1.153
Self-care status (self-care)	−0.145	0.727	−0.013	−0.200	0.842	−1.578 ~ 1.288	1.139

R^2^ = 0.331, adjusted R^2^ = 0.289, F = 7.844, *p* < 0.001.

Categorical variable coding: sex (0 = female, 1 = male); educational level (reference: primary school and below); marital status (0 = no spouse, 1 = having a spouse); emergency admission (0 = no, 1 = yes); primary caregiver (0 = dependent on caregivers, 1 = self-care). Bold values indicate statistically significant results (*p* < 0.05).

For utilization of social support (Table 6), higher insight level (ITAQ *β* = 0.141, 95% CI: 0.100 ~ 0.182, *p* < 0.001) and psychiatric symptom severity (BPRS *β* = 0.028, 95% CI: 0.002 ~ 0.055, *p* = 0.037) were positively associated, whereas higher suicide risk (NGASR: *β* = −0.163, 95% CI: −0.277 ~ −0.049, *p* = 0.005) was negatively associated (adjusted *R*^2^ = 0.194).

**Table 6:** Linear regression analysis of utilization of social support in different schizophrenia patients (*n* = 204).

Variables	Non-standardized *β*	Coefficients standardized error	Standardized coefficients beta	*t*	*p*	95% CI	VIF
Constant	3.097	1.413	—	2.192	**0.030**	0.311 ~ 5.884	—
Gender	−0.526	0.326	−0.107	−1.611	0.109	−1.170 ~ 0.118	1.100
Age	0.009	0.019	0.032	0.473	0.637	−0.028 ~ 0.046	1.145
Course of schizophrenia	−0.017	0.011	−0.111	−1.642	0.102	−0.038 ~ 0.004	1.139
Baseline disease severity (BPRS)	0.028	0.014	0.149	2.104	**0.037**	0.002 ~ 0.055	1.250
Educational level (Middle school)	0.548	0.354	0.121	1.550	0.123	−0.149 ~ 1.246	1.531
Educational level (Senior high school and above)	0.251	0.359	0.056	0.699	0.485	−0.457 ~ 0.959	1.589
ITAQ (levels of insight)	0.141	0.021	0.489	6.749	**<0.001**	0.100 ~ 0.182	1.318
NGASR (risk of suicide)	−0.163	0.058	−0.212	−2.829	**0.005**	−0.277 ~ −0.049	1.409
GDS (levels of depression)	−0.001	0.021	−0.003	−0.033	0.974	−0.043 ~ 0.041	1.525
Marital status (have a spouse)	0.064	0.310	0.014	0.207	0.837	−0.547 ~ 0.675	1.197
Emergency admission (Yes)	−0.126	0.293	−0.029	−0.429	0.669	−0.704 ~ 0.453	1.153
Self-care status (self-care)	−0.371	0.372	−0.067	−0.997	0.320	−1.104 ~ 0.362	1.139

R^2^ = 0.242, adjusted R^2^ = 0.194, F = 5.063, *p* < 0.001.

Categorical variable coding: sex (0 = female, 1 = male); educational level (reference: primary school and below); marital status (0 = no spouse, 1 = having a spouse); emergency admission (0 = no, 1 = yes); primary caregiver (0 = dependent on caregivers, 1 = self-care). Bold values indicate statistically significant results (*p* < 0.05).

## 4 Discussion

This study investigated the level of social support and its associated factors among older inpatients with schizophrenia. The mean SSRS score of 30.26 was lower than that reported in community-dwelling older adults in Shanghai (38.29 ± 5.2) (24) but comparable to other inpatient schizophrenia samples (33.39 ± 6.65) (25). Regression analyses identified distinct patterns across SSRS dimensions, with insight, marital status, suicide risk, depressive symptoms, and disease duration emerging as independently associated factors.

Consistent with previous research (17), insight level (ITAQ) was positively associated with social support across all four SSRS dimensions. Patients with better insight likely engage more effectively in therapeutic relationships and communicate their needs more clearly, thereby facilitating the receipt and perception of social support (26). Better insight may also enable patients to articulate their needs to caregivers and healthcare providers, mobilizing tangible assistance in the hospital setting. For subjective support, greater insight may mitigate the emotional dissonance between self-perception and external feedback, thereby reducing anxiety-driven social withdrawal and enabling more accurate perception of available support (27). Regarding support utilization, higher insight was associated with more active help-seeking; patients with greater insight appeared to engage more effectively in shared understanding during clinical interactions, which may improve treatment adherence and strengthen trust in the support system (28). These findings suggest that insight-oriented interventions, such as cognitive-behavioral therapy or motivational interviewing, should be integrated into routine inpatient care and evaluated in future interventional studies for their potential to strengthen social support in this vulnerable population.

Having a spouse was consistently associated with higher total SSRS, objective support, and subjective support, consistent with prior evidence that married patients with schizophrenia report greater social support (13). This protective effect persisted despite hospitalization, suggesting that the marital bond remains a robust source of both tangible assistance and emotional reassurance even during physical separation (29). Clinically, these findings underscore the importance of involving spouses in psychoeducation and discharge planning (30). For patients without spouses, healthcare providers should identify alternative stable support figures, such as adult children, siblings, or close friends, and actively facilitate their involvement in care planning.

In contrast to these protective factors, emotional distress was associated with diminished social support across multiple dimensions. Our results demonstrated that higher suicide risk (NGASR) was negatively associated with total SSRS, subjective support, and support utilization, while greater depressive symptoms (GDS) were negatively associated with total SSRS, objective support, and subjective support. These findings are consistent with meta-analytic evidence that social support protects against suicide-related behaviors in severe mental illness (31). Notably, subjective support exhibited stronger associations with suicide risk than objective support, suggesting that perceived emotional support, through fostering belonging, self-worth, and hope, may be more protective against suicidality than tangible assistance (32). The negative association between depressive symptoms and objective support may partly reflect the inpatient context, where institutional care replaces some informal support while the restrictive ward environment limits access to external social networks. These findings suggest that suicide risk and depressive symptoms undermine social support through related but dissociable pathways: suicide risk predominantly impairs support utilization, whereas depressive symptoms more broadly erode objective support. Routine screening for both conditions should be integrated into psychosocial assessment, and at-risk patients may benefit from interventions concurrently addressing mood symptoms and support mobilization, such as cognitive-behavioral therapy or peer support programs.

Longer disease duration and self-caring status were also independently associated with lower social support, albeit through different dimensions. An important finding of this study is that self-caring patients reported significantly lower objective social support than those who depended on others for daily care. Although clinically intuitive, this pattern has rarely been empirically demonstrated in older inpatients with schizophrenia. Objective support encompasses tangible assistance such as material aid and practical care, which self-caring patients are less likely to receive in the hospital setting. In China, the family remains the predominant source of objective support for older adults (33), and a recent qualitative study highlighted that older schizophrenia patients without caregivers experience extreme social isolation and difficulty accessing welfare resources (34). For such patients, healthcare systems and social services should assume greater responsibility in providing structured support, such as assigning case managers or integrating peer support programs into inpatient care. Additionally, longer disease duration was independently associated with lower total SSRS and subjective support, reflecting progressive erosion of social networks over the illness trajectory (35). This finding underscores the need for early intervention programs that help patients maintain community connections, particularly during the transition from inpatient to community care.

## 5 Limitations

Several limitations should be acknowledged. First, the cross-sectional design precludes causal inference; the observed associations may be bidirectional, and longitudinal studies are needed to clarify temporal relationships. Second, cognitive function was not formally assessed. This is an important limitation because cognition may influence responses to the self-reported measures (SSRS, GDS-30) and could also affect the interviewer-rated assessments (ITAQ, NGASR and BPRS) administered by trained research assistants. Future studies should therefore include a standardized cognitive assessment (e.g., MMSE or MoCA) to evaluate the potential impact of cognitive function on these measures. Third, marital status was dichotomized due to sample size constraints, and several potential confounders, including symptom subtypes, socioeconomic status, and pre-hospitalization community support, were not measured. The SSRS, though widely used in Chinese populations, requires further validation in hospitalized patients. Finally, this single-center study in a tertiary psychiatric hospital may limit generalizability; multi-center studies across different regions and hospital levels are warranted.

## 6 Conclusion

In summary, this study found that social support among older inpatients with schizophrenia was relatively low and associated with multiple demographic, clinical, and psychological factors. Having a spouse and higher insight were consistently associated with better social support, whereas higher suicide risk, greater depressive symptoms, longer disease duration, and self-caring status were negatively associated. Notably, self-caring patients reported significantly lower objective support, highlighting a vulnerable subgroup that may be overlooked in routine care. These findings suggest that involving spouses in psychoeducation and discharge planning, while identifying alternative support figures for unmarried patients, may help sustain social support. Insight-oriented interventions, suicide risk reduction, and depression management may also benefit support perception and utilization. Future longitudinal and multi-center studies are needed to clarify causal mechanisms and strengthen generalizability.

## Data Availability

The raw data supporting the conclusions of this article will be made available by the authors, without undue reservation.
